# *Babesia microti* Infection Changes Host Spleen Architecture and Is Cleared by a Th1 Immune Response

**DOI:** 10.3389/fmicb.2018.00085

**Published:** 2018-01-31

**Authors:** Vitomir Djokic, Lavoisier Akoolo, Nikhat Parveen

**Affiliations:** Department of Microbiology, Biochemistry and Molecular Genetics, Rutgers New Jersey Medical School, Newark, NJ, United States

**Keywords:** *Babesia microti*, protozoan pathogenesis, babesiosis, tick-borne infection, immunosuppression, blood-borne pathogen

## Abstract

*Babesia microti* is a malaria-like parasite, which infects ∼2000 people annually, such that babesiosis is now a notifiable disease in the United States. Immunocompetent individuals often remain asymptomatic and are tested only after they feel ill. Susceptible C3H/HeJ mice show several human-like disease manifestations and are ideal to study pathogenesis of *Babesia* species. In this study, we examined parasitemia of *B. microti* at different time points and assessed its impact on hemoglobin levels in blood, on spleen pathology and overall immune response in C3H/HeJ mice. Peak parasitemia of 42.5% was immediately followed by diminished hemoglobin level. Parasitemia at 21 days of infection was barely detectable by microscopy presented 5.7 × 10^8^ to 5.9 × 10^9^ *B. microti* DNA copies confirming the sensitivity of our qPCR. We hypothesize that qPCR detects DNA released from recently lysed parasites or from extracellular *B. microti* in blood, which are not easily detected in blood smears and might result in under-diagnosis of babesiosis in patients. Splenectomized patients have been reported to show increased babesiosis severity and result in high morbidity and mortality. These results emphasize the importance of splenic immunity in resolution of *B. microti* infection. Splenomegaly in infected mice associated with destruction of marginal zone with lysed erythrocytes and released *B. microti* life forms in our experiments support this premise. At conclusion of the experiment at 21 days post-infection, significant splenic B and T cells depletion and increase in macrophages levels were observed in *B. microti* infected mice suggesting a role of macrophage in disease resolution. Infected mice also showed significantly higher plasmatic concentration of CD4 Th1 cells secreted cytokines such as IL-2 and IFN-γ while cytokines such as IL-4, IL-5, and IL-13 secreted by Th2 cells increase was not always significant. Thus, Th1 cells-mediated immunity appears to be important in clearance of this intracellular pathogen. Significant increase in IL-6 that promotes differentiation of Th17 cells was observed but it resulted in only moderate change in IL-17A, IL-17F, IL-21, and IL-22, all secreted by Th17 cells. A similar immune response to Trypanosoma infection has been reported to influence the clearance of this protozoan, and co-infecting pathogen(s).

## Introduction

Tick-borne diseases have been increasing steadily in the last two decades in North America and Europe that include bacterial, viral and parasitic pathogens. Transmission of multiple pathogens by a single tick bite has been reported to occur (Dunn et al., 2014; Rizzoli et al., 2014; Moutailler et al., 2016). *Babesia* species are tick-transmitted parasites with the most cases of babesiosis attributed to *Babesia microti* in the United States, with some patients show infection with *B. duncani* in Western states, while *B. divergens* is primarily responsible for babesiosis in Europe (Centers for Disease Control and Prevention, 2012). Approximately 2000 cases of babesiosis occur in the United States every year, which resulted in its declaration as a notifiable disease by CDC in 2011. *B. microti* infection affects the Northeastern United States; Connecticut, Massachusetts, New Jersey, New York, and Rhode Island along with the Midwestern states of Wisconsin and Minnesota (Knapp and Rice, 2015). Together they represented 94% of babesiosis cases in the United States in 2014.

*Babesia* species are intracellular parasites of red blood cells (RBCs), where they multiply until erythrocytes burst. Clinical disease ranges from asymptomatic or mild flu-like symptoms in immunocompetent people, including fever and myalgia, to acute or sometimes fatal disease in immunocompromised or splenectomized individuals (Genda et al., 2016). Patients are often tested for babesiosis only after observation of hemolytic anemia. *Babesia* species can also transmit through transplacental route and can cause jaundice, anemia and neutropenia in children (Joseph et al., 2011, 2012; Luckett et al., 2014). Immunocompetent individuals can establish *Babesia* carriage state without any clinical manifestations for long periods of time. Due to survival of these parasites during cold storage, infected donated whole blood and blood products can cause blood transfusion transmitted babesiosis (TTB) that have severe consequences in the immunocompromised patients (Herman et al., 2010; Herwaldt et al., 2011; Sinski et al., 2011; Cushing and Shaz, 2012; Cursino-Santos et al., 2014; Fang and McCullough, 2016). Babesiosis is the most prevalent transfusion transmitted disease in the United States such that FDA has recently recommended that blood donors should be screened for *Babesia* infection (Lobo et al., 2013; United States Food and Drug Administration, 2014).

Current confirmatory test for babesiosis is microscopic examination of Giemsa-stained blood smears. However, this method is time consuming and requires a specific expertise for correct diagnosis because pleomorphic, non-synchronous trophozoites and ring forms can make *Babesia* identification difficult. This method is also very labor intensive. Therefore, it cannot be used for large scale testing of donated blood samples. Some diagnostic laboratories employ FISH for diagnosis of babesiosis that also requires microscopic examination. Serologic tests are more sensitive and efficient; however, these tests cannot detect acute disease before the adaptive immune response is established and are also unable to distinguish active disease from the past infections or reinfections, thus, creating a major diagnostic problem particularly in the endemic regions (Magnarelli et al., 1998; Foppa et al., 2002; Hunfeld et al., 2002; Yoshinari et al., 2003; Fox et al., 2006; Vannier et al., 2008; Lempereur et al., 2015; Mayne, 2015; Wormser et al., 2015). Nucleic Acid Tests have been used for examination of both humans and ticks and have more promise for diagnosis of babesiosis including blood donors (Bose et al., 1995; Krause et al., 1996a; Cushing and Shaz, 2012; Michelet et al., 2014; Jahfari et al., 2016). We found that our, molecular beacon probes based real-time quantitative Polymerase Chain Reaction (qPCR) test, identifies patient samples with babesiosis with greater sensitivity, in a tick-borne diseases endemic state, New Jersey.

White footed mouse is natural reservoir of *B. microti* in the United States. Susceptible C3H mice show several manifestations like humans and offer ideal animal model system to investigate pathogenesis of this protozoan parasite (Ruebush and Hanson, 1979). Observation of pronounced splenomegaly in *B. microti* infected C3H/HeN mice as well as higher parasitemia levels and delayed clearance of *B. microti* from blood stream of splenectomized mice indicate importance of spleen immunity in resolution of babesiosis even in mice (Coleman et al., 2005). *B. microti* parasitemia also reduced in Severe Combined Immunodeficient (SCID) mice by adoptive transfer of splenocytes from naïve immunocompetent mice, further emphasizing the role of adaptive immune response facilitated by splenic cells (Vannier et al., 2004). However, a systematic study to assess the consequence of *B. microti* infection on mice together with both plasmatic and splenic immunity has not been carried out until now. Therefore, *B. microti* pathogenesis and immunological response to this intraerythrocytic pathogen remain poorly understood. Involvement of innate immunity determined by macrophage during *B. microti* infection was suggested to play the primary role in babesiosis resistance in mice (Aguilar-Delfin et al., 2001; Terkawi et al., 2015). Adoptive transfer of primed T cells was reported to control mice reinfection by *B. microti* likely due to also simulating primed B cell response (Meeusen et al., 1985). Furthermore, CD4 and not CD8 cells were shown to participate in elimination of *B. microti* in mouse infection model (Hanafusa et al., 1998; Igarashi et al., 1999; Hemmer et al., 2000a). Interestingly, human *Babesia* WA1 parasites infection in mice resulted in upregulation of both TNFα and IFNγ resulting in severe pathogenesis and fatal disease (Hemmer et al., 2000b). Surprisingly, anti-IFNγ monoclonal antibodies partially reduced protection of mice immune against *B. microti* and IFNγ-deficient mice did not offer any protection against *B. microti* infection (Igarashi et al., 1999).

We followed the multiplication cycle of *B. microti* in blood of C3H/HeJ mice and assessed the impact of parasitemia increase on hemoglobin levels in blood, on spleen pathology and on overall splenic and plasmatic immune response. Our studies demonstrate that innate immune response particularly due to macrophage participation and adaptive immune response triggered by stimulation of CD4 cells play critical roles in elimination of parasitized erythrocytes during infection of mice with *B. microti*. Furthermore, we also describe potential reason for under detection of *B. microti* during human infection.

## Materials and Methods

### Human Samples

Blood samples from 133 patients collected in 2015 from three different counties in New Jersey as described previously were used in this study (Akoolo et al., 2017). Patients presenting different clinical symptoms were recommended for testing for tick borne diseases for initial evaluation, or follow-up care. IGeneX tested air-dried blood smears for babesiosis by FISH using 18S rDNA/rRNA as target. Stony Brook Laboratory for Lyme disease performed serological testing by Indirect IFA. Blood testing for babesiosis was conducted by microscopic examination of Giemsa-stained smears. Remnant, coded sample aliquots (collected for their own testing) were provided for another study to the corresponding author. Results of their tests were provided in unidentifiable/anonymized manner under corresponding author’s approved, exempt-Institutional Review Board (e-IRB) protocol, Pro2013002634, approved by Institutional Review Board of Rutgers New Jersey Medical School.

### *B. microti* Infection Cycle in C3H/HeJ Mice

The Newark Institutional Animal Care and Use Committee (IACUC) designated members reviewed and approved the protocol number D-14011-A1 under which this study was conducted at Rutgers University New Jersey Medical School following guidelines of the Animal Welfare Act, The Institute of Laboratory Animal Resources Guide for the Care and Use of Laboratory Animals, and Public Health Service Policy. *B. microti* (ATCC30221) was first propagated in SCID mice to obtain inoculum for subsequent experiments because these parasites adapted to a particular host allow manifestation of full spectrum of pathogenesis of *B. microti* (Gumber et al., 2016). Ten 4 weeks old C3H/HeJ mice were infected at a dose of 10^4^ parasitized RBCs/mouse. To determine parasitemia, 5–10 μl blood was collected from each infected mouse by tail bleed to prepare blood smears that were Giemsa-stained according to CLSI guidelines (Garcia et al., 2000). On the day of infection experiment in immunocompetent C3H/HeJ mice, blood was collected from SCID animals and inoculum dose determined. Briefly, total erythrocytes per milliliter of blood were determined using a hemocytometer and parasitized RBCs counted in a total of 20–50 fields at high magnification. Numbers of infected RBCs were determined by multiplying percent parasitemia with the total count of RBCs/ml of blood. The number of parasitized RBCs was then adjusted to 10^5^/ml in PBS and 100 μl suspension injected in each mouse intraperitoneally such that infection dose of 10^4^ infected erythrocytes per animal was used to infect 4 weeks old C3H/HeJ mice. Five uninfected mice were included as controls for all analyses.

### Monitoring of Parasitemia in Blood of Infected Mice

Blood smears from the infected mice were prepared, and Giemsa stained. Blood smears were then examined to determine parasitemia and thus, progression of *B. microti* infection up to 21 days of infection. Blood hemoglobin levels were determined using a commercial kit (Hemocue^®^ Hb 201+ analyzer, Sweden) according to the manufacturer’s instructions. Mice were euthanized at 21 days post infection and evaluated further for disease pathology. Before euthanasia, heparinized blood was collected from each mouse by cardiac puncture and then mice were euthanized. Plasma was recovered after centrifugation for cytokine profile determination.

### IFA to Detect *B. microti* in Blood and Spleen

For paraffin embedded spleen sections, slides were immersed in xylene two times for 10 min interval to remove paraffin and was followed by immersion in 100% ethanol twice and then immersion in 95, 70, and 50% ethanol for 5 min each. A final rinse with water was followed by rehydration in PBS. Slide edges were marked with hydrophobic marker and then IFA was conducted as described previously using pooled patient sera from four individuals that tested positive in our previous studies (Akoolo et al., 2017). Staining with 2-(4-amidinophenyl)-1H-indole-6-carboxamidine (DAPI) was included to stain nucleus with the anti-human secondary antibody conjugated to Alexa fluor 488 (Molecular Probes).

### Quantification of *B. microti* in Blood by qPCR

DNA was isolated from mice blood using QIAamp Blood midi kit (Qiagen). Duplex qPCR was conducted using different dilutions of genomic using the primers and molecular beacon probe for gene encoding Thiamine pyrophosphokinase (*B. microti TPK*) as we described previously (Chan et al., 2013; Akoolo et al., 2017). Amplification was performed in 25 μl reaction mixtures. Based upon the genome size (6.5 Mb), of *B. microti*, 8 ng of DNA was calculated to contain 10^6^ copies of *B. microti* TPK gene. A standard curve was prepared using 10-fold dilutions of *B. microti* genomic DNA. Leukocytes from mouse blood were used to amplify Nidogen gene as an internal control. Inclusion of this control ensured that the quality of DNA was suitable for PCR (Chan et al., 2013).

### Isolation of and Characterization of Splenocytes by Flow Cytometry

Aseptically harvested spleens from infected and uninfected mice were imaged, weighed and single cell suspension of the splenocytes prepared. Briefly, spleen was sliced into small pieces and strained through a 70 μm nylon sterile cell strainer into 50 ml conical tube. After washing cells with Phosphate buffered saline by centrifugation at 1,500 rpm, RBCs were lysed using Ammonium-Chloride–Potassium lysis buffer (Thermo Fisher). The splenocytes were then resuspended in FACS buffer at 10^6^/ml concentration and stained by labeling with respective marker antibodies tagged with different fluorophores. For staining, the cells were incubated for 30 min in the dark at 4°C using the fluorophores tagged antibodies; B cells with Brilliant violet 421 anti-mouse CD19 antibody (Biolegend), NK cells with APC-Cy7 anti-mouse NK1.1 antibody (Bilegend), CD3 with PE-Cy7 anti-mouse CD3 antibodies (Biolegend), and macrophages with anti-mouse F4/80 Brilliant violet 605 antibody. The cells were washed with PBS containing 2% FBS by centrifugation, and resuspended in Fluorescence Activated Cell Sorting (FACS) buffer. Cells were sorted using a flow cytometer BD LSRFortessa^TM^ X-20 (BD Biosciences) driven by software FACS DiVa (BD Biosciences). Analysis of the acquired data was performed using software, FlowJo, Version 10.3.

### Determination of Plasmatic Cytokines Changes in the Infected Mice

Heparinized blood samples were centrifuged and plasma was collected. Major cytokines were detected and quantified using commercial bead-based immunoassay kit LEGENDplex^TM^, mouse multiplex cytokine panel (Biolegend) according to manufacturer’s instructions with cytokines IFN-γ, TNF-α, IL-2, IL-4, IL-5, IL-6, IL-10, IL-13, IL-17A, IL-17F, IL-21 and IL-22 analyzed. Briefly, all plasma samples were diluted in 1:1 in assay buffer and all analyses were performed in duplicates for each sample. Samples were treated according to Biolegned’s standard protocol, washed using the filter plates, and then examined using the BD LSRFortessa^TM^ X-20 (BD Biosciences) driven by software FACS DiVa (BD Biosciences). Calculations were performed using Legendplex 7.1 application (Vigene Tech Inc.).

### Statistical Analysis

All data collected was analyzed by GraphPad Prism 7.0a (GraphPad Software). Results are presented as mean ± standard deviation (S.D.). Comparisons were made between groups using unpaired student two-tailed *t*-tests for unequal variance to determine statistical significant difference between paired groups. Thus, values below 0.05 were considered significant for a paired group comparison at 95% confidence interval.

## Results

### *B. microti* Infection Is Detected More Efficiently by qPCR Than IFA, FISH, or Blood Smear Microscopy

**FIGURE 1:**
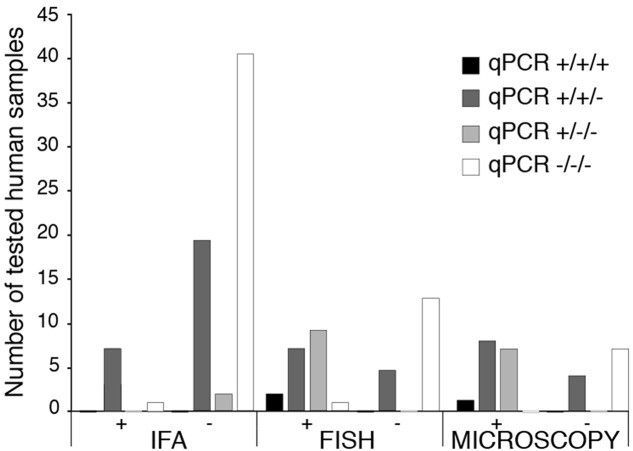
Comparison of diagnostics by IFA, FISH and Microscopy for babesiosis with qPCR using molecular beacon probes for *BmTPK* in human blood samples. For all patients samples, qPCR was repeated three times and results of each repeat are marked by ‘+’ or ‘–’ sign. Thus, +/+/+ indicates that qPCR was positive in all three repeats of the qPCR for an individual sample. There is a high correlation between all three tests when samples were negative by qPCR. Higher sensitivity of detection of *B. microti* by qPCR is demonstrated by a significant number of positive samples that were diagnosed negative for babesiosis by Indirect IFA, FISH and microscopic examination of Giemsa-stained blood smears.

All patient samples were tested three times and are shown as positive (+) or negative (-) for each test. Thus, (+++) depict that assay was positive all three times while (+) indicates one positive out of three repeats, likely due to low parasitemia. Out of 133 human tested samples, qPCR detected 71 positive and 62 negatives. Seven samples were positive by both qPCR and IFA, 16 by qPCR and FISH, and 15 by qPCR and microscopic examination of Giemsa-stained blood smears (**Figure 1**). We detected 21 qPCR positive samples that were negative by IFA, five that were FISH negative, and four in which parasites-infected erythrocytes were not detected by microscopy (**Figure 1**). Almost all qPCR negative samples (60/62) were negative in other diagnostic tests. Only two samples that were positive by IFA and FISH produced negative qPCR results (**Figure 1**). Thus, our qPCR could detect *B. microti* presence in blood in a more sensitive manner than other currently used tests.

### Parasitemia Pattern in Mice Detected by Microscopy

**FIGURE 2:**
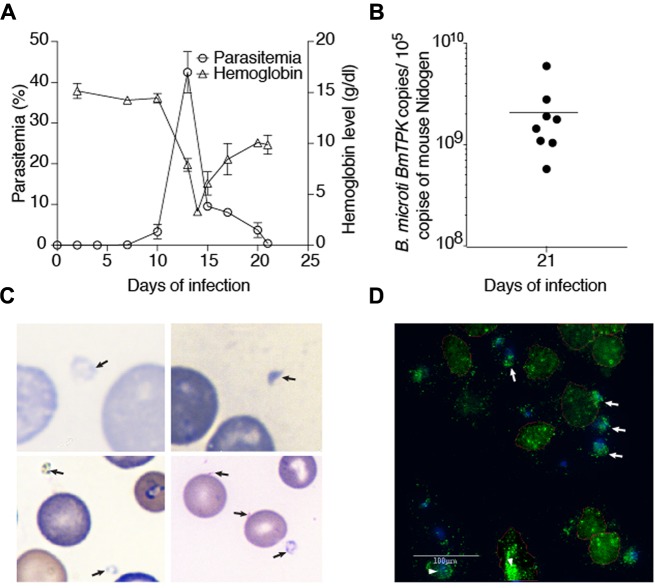
Course of *B. microti* infection and detection of parasites in blood of susceptible C3H/HeJ mice. **(A)** Increase in parasitemia resulted in a dramatic drop in hemoglobin levels reaching the lowest levels 1 day after peak *B. microti* parasitemia was obtained. Recovery of hemoglobin was rapid after decline in parasitemia. Parasitized erythrocytes were barely detectable by microscopy on 21st day of infection. **(B)** High copy numbers of *B. microti* DNA were present in blood of the infected animals at this stage of infection. **(C)** Several extracellular, released parasites can be observed in Giemsa stained blood smear of infected mice supporting our qPCR results. **(D)** The presence of external, released green fluorescent *B. microti* (marked by arrows) as well as intracellular parasites (marked by arrowheads) were observed by IFA of the blood smears confirming Giemsa stained blood smears results shown in **(C)**.

We inoculated ten C3H/HeJ mice with *B. microti* to study its pathogenesis. Seven days after infection, 0.1% parasitemia was obtained that steadily increased and culminated to its peak level of average 42.5% on 13th day of infection (**Figure 2A**) and then started declining. Increased *B. microti* parasitemia was followed by depletion of RBCs and decreased hemoglobin. One day after observed peak parasitemia, i.e., on day 14 of infection, hemoglobin levels reached to the minimal levels of 3.3 g/dl. Hemolytic anemia is also a hallmark of symptomatic babesiosis in humans. With subsequent decline in parasitemia, hemoglobin levels started returning to normal values with the value of 9.8 g/dl detected on 21st day post infection (**Figure 2A**). At this time point, parasitemia was barely detectable (<0.03%) by microscopic examination of Giemsa-stained blood smears. We then performed qPCR to assess the presence of the parasitic DNA in blood at this stage of infection because our qPCR was found to be more sensitive in detection of *B. microti* presence in the human blood (**Figure 1**). Our results with mouse samples confirmed the sensitivity of the qPCR test because surprisingly, high parasitic DNA copy numbers of 5.7 × 10^8^ to 5.9 × 10^9^ were detected in different infected mice at this stage (**Figure 2B**). To identify cause of this discrepancy between two tests, and determine whether persistence of *B. microti* DNA in blood was responsible for our qPCR results, we further examined blood smears more carefully and identified several *B. microti* structures outside the erythrocytes (**Figures 2C,D**). Detection of these parasites explains release of parasites from lysed RBCs and corroborates our qPCR results. It is possible that these released parasites failed to infect RBCs due to significantly reduced levels of mature erythrocytes in blood (Borggraefe et al., 2006; Skariah et al., 2017). The lack of detection of *B. microti* DNA in the control mice was as expected (data not shown).

### Effect of *B. microti* Infection on Spleen of Mice

**FIGURE 3:**
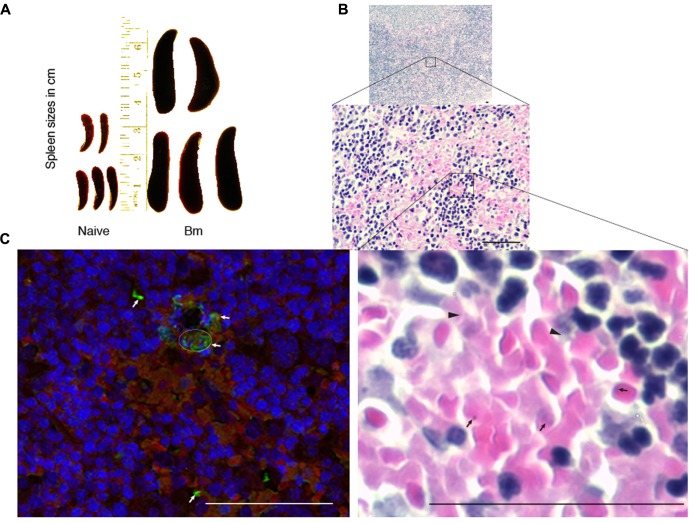
Impact of *B. microti* infection on spleen of the infected mice. **(A)** *B. microti* infection resulted in a significant increase in spleen sizes of mice compared to uninfected, naïve mice. **(B)** White and red pulp zones were significantly enlarged while demarcation zone was not apparent in the spleen of *B. microti* infected mice. Numbers of lysed erythrocytes (marked by arrowheads) as well as various free parasitic forms (marked by arrows) were also observed in infected mouse spleen. **(C)** In IFA conducted the spleen section, red color indicates auto-fluorescence of RBCs, blue shows nuclear staining of cells while green fluorescence marks *B. microti* probed with infected human plasma followed by detection with Alexa fluor 488 conjugated secondary antibodies. Apparent lysed infected erythrocytes presence is marked by a circle. Several free, released parasitic forms were also detected (marked by arrows) among erythrocytes. Bars represent 25 μm.

Spleen is considered to play an important role during babesiosis in patients such that splenectomized patients show more severe life-threatening disease. Systematic analysis of contribution of spleen in babesiosis resolution in humans is not possible. Therefore, we decided to study the impact of *B. microti* infection on spleen of infected mice. Spleen sizes of *B. microti* infected mice consistently increased, i.e., significantly higher than that of the spleen sizes of the naïve mice, as described previously (Coleman et al., 2005). Comparative sizes of infected and naïve mice, five mice each, are shown (**Figure 3A**). Weights of spleen correlated with their increased size in the infected mice (average weight 0.743 g) as compared to naïve animals average weight 0.08 g) and difference was statistically significant (*p* < 0.0001, 95%CI). Histopathological examination of spleen sections after Hematoxylin-Eosin staining showed depletion of marginal zone in *B. microti* infected animals. In addition, enlarged red and white pulps were observed in the spleens of the infected mice such that these two zones were found interwoven throughout the organ (**Figure 3B**). A careful examination of spleen sections under high magnification also showed the presence of lysed RBCs as well as extracellular *B. microti* life forms (**Figure 3B**). The presence of the released, extracellular parasites in spleen was further confirmed by IFA (**Figure 3C**). Spleen and liver are the major organs of reticuloendothelial system. This system plays a critical role in degradation of defective erythrocytes and cleaning the blood impurities. In our experiments, mouse spleen appears to play a role in clearance of parasitized RBCs. It is possible that another organ of this system, liver, could also play a role in removal of *B. microti* infected RBCs.

### Effect of *B. microti* Infection on Splenic Immune Cells

We further examined whether there are changes in splenocytes involved in immunity to allow clearance of infected RBCs. Surprisingly, *B. microti* infected mice on day 21 post infection showed difference in numbers of splenic B, T cells, and macrophages in comparison with naïve animals. Total cell count from each mouse for FACS analysis was adjusted to 100,000/per mouse. Significant depletion of both CD19+ B cells from an average of total 39,655 (39.6%) in naïve to 16,119 (16.3%) in *B. microti* infected mice, and from total average 18,423 (18.42%) CD3+ T cells in control group to 10.1% in *B. microti* infected mice were observed consistently. These observations indicate that *B. microti* infection causes significant subversion of adaptive immune response determine by spleen. Interestingly, macrophages (F4/80) levels were higher with total average of 6,641 (6.6%) macrophage in mice infected with *B. microti* as compared to an average of 1,892 (1.9%) in control animals. Splenic NK1.1 cells total count and percentage remained unaffected after *B. microti* infection 20,781versus 20,615 (20.8% versus 20.6%), respectively. These results indicate a major role of innate immune response in clearance of parasites. Increase in splenic macrophage suggests their contribution in clearance of parasitized erythrocytes that reach spleen as an organ in reticuloendothelial system. Although no changes in splenic NK1.1 cell percentage were observed, their role in resolution of parasitemia cannot be ruled out. Enlargement and dark color of spleen of *B. microti* infected mice could be due to increased hematopoietic support and increased macrophages activity resulting in erythrophagocytosis.

### Detection of Mouse Pro-inflammatory Cytokines in Plasma

**FIGURE 4:**
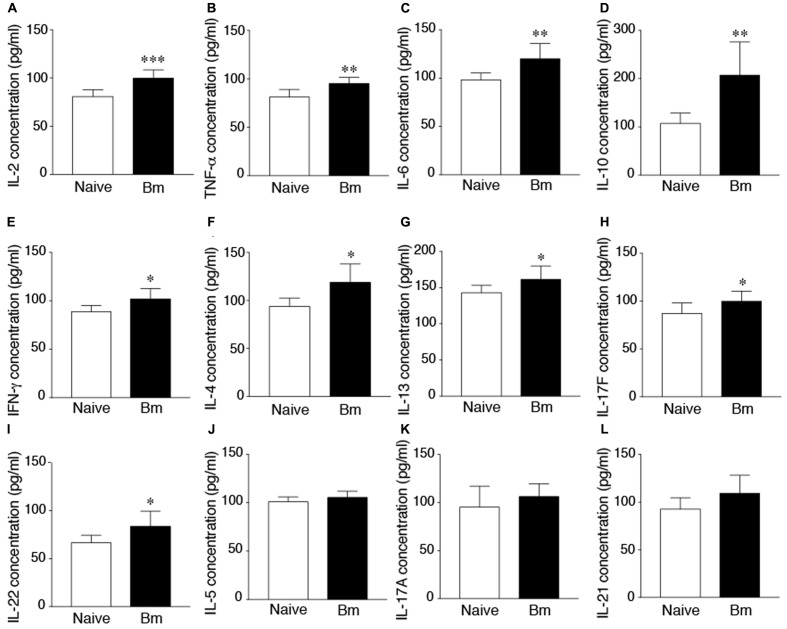
Increased cytokine levels in mice after 21 days of infection. **(A)** Highest difference between *B. microti* infected and uninfected, naïve mice was observed in concentration of IL-2 (99.7 pg/ml compared to 80 pg/ml, *p* < 0.0005). **(B–D)** Concentrations of TNF-α (95.2 pg/ml), IL-6 (119.8 pg/ml) and IL-10 (207.1 pg/ml) were significantly higher in infected mice relative to control group (*p <* 0.005, *<*0.01, <0.01, respectively). **(E–I)** IFN-γ, IL-4, IL-13, IL-17F, and IL-22 of *B. microti* infected mice, showed modest but statistically significant increase in infected mice plasma relative to the naïve animals (*p <* 0.05 each; 95% CI). **(J–L)** Concentrations of IL-5, IL-17A, and IL-21 were not significantly different between infected and uninfected mice. Comparisons were made between groups using unpaired student two-tailed *t*-tests for unequal variance at 95% Confidence Interval (CI) (^∗^*p* < 0.05, ^∗∗^*p* < 0.01, ^∗∗∗^*p* < 0.005).

The cytokine superfamily of proteins is involved in the signaling and communications between cells. They are fingerprints of the immune response generated in response to infection, injury or even cancer. Plasmatic cytokine profile is more important for clearance of blood borne pathogens. Therefore, we decided to determine changes in immune response in blood by analysis of plasmatic cytokines profile in response to *B. microti* infection. We examined the levels of 13 cytokine levels in plasma of *B. microti* infected versus uninfected mice. *B. microti* infection did not affect level of IL-9 in mouse plasma (data not shown). IFN-γ, TNF-α, IL-2, IL-4, IL-6, IL-10, IL-13, IL-17F, and IL-22, showed significantly higher levels compared to naïve animals (**Figure 4**). Highest differences were observed for IL-2 of *B. microti* infected mice (99.7 pg/ml) compared to naïve animals (80.8 pg/ml) (**Figure 4A**). *B. microti* infected mice also displayed higher concentrations of TNF-α (95.2 pg/ml), IL-6 (119.8 pg/ml) and IL-10 (207.1 pg/ml) which were all significantly higher than the control group displaying concentrations of 81.8, 98.2, 107 pg/ml, respectively (**Figures 4B–D**). IFN-γ, IL-4, IL-13, IL-17F, and IL-22 in *B. microti* infected mice showed concentrations of 101.9, 118.9, 161.3, 99.7, 83.7 pg/ml, respectively, relative to the naïve animals that showed concentrations of 88.8, 93.8, 143, 87, 66.4 pg/ml, respectively (**Figures 4E–I**). Difference between IL-5, IL-17A, and IL-21 levels between *B. microti* infected and uninfected mice were not significant (**Figures 4J–L**). Considering cytokines have a short life, the changes in cytokine profile here could represent an ongoing low level *B. microti* infection in mice that could not be detected in blood smears but was detectable by qPCR. Furthermore, a declining cytokine levels are likely detected at this stage of infection such that potential higher levels of these cytokines presence during peak parasitemia to allow parasite clearance.

## Discussion

*Babesia microti* infection of humans has become very prominent in the last decade, both in North America and Europe (Hersh et al., 2014; Rizzoli et al., 2014; Knapp and Rice, 2015; Diuk-Wasser et al., 2016). In majority of immunocompetent individuals, infection remains unseen, with mild, flu-like symptoms or diminished hemoglobin levels and anemia. However, in combination with other tick-transmitted diseases such as *Borrelia burgdorferi* even in healthy person this parasite can intensify clinical disease and its severity (Krause et al., 1996b; Knapp and Rice, 2015). Babesiosis has tremendous consequences in the immunocompromised individuals and infants and can even be fatal (Usmani-Brown et al., 2013; Menis et al., 2015; Akoolo et al., 2017). Equally dangerous is newly identified route of transmission of *B. microti*, via blood transfusion. In these cases, immunocompetent donors are undiagnosed and remain *Babesia* carriers such that blood recipients who are often immunocompromized individuals can exhibit high morbidity and mortality (Herwaldt et al., 2011; Cushing and Shaz, 2012; Kitt et al., 2016).

Several diagnostic tests are available for diagnosis of babesiosis; however, blood smear test is still considered gold standard by CDC and WHO (Clinical and Laboratory Standards Institute, 2000; Akoolo et al., 2017). One reason is that antibodies can persist even more than a year after the clearance of *Babesia* infection (Vannier and Krause, 2012). On the other hand, immunocompromised or immunodeficient individuals can remain seronegative, although they could be carrier of infectious parasites, in some cases for more than 2 years (Raffalli and Wormser, 2016). PCR, as a diagnostic test for *B. microti* infection, was first described by Persing et al. (1992), which could detect as little as three merozoites per microliter of blood (Persing et al., 1992). Since then, method has been improved greatly. Our laboratory in 2013 developed multiplex PCR method based on molecular beacons probe for *BmTPK* amplicon, which significantly improved specificity and sensitivity of the assay (Chan et al., 2013) We later also employed this test to examine human samples from endemic zone for tick-borne diseases (Akoolo et al., 2017). For majority of human samples, we were unable to determine time of the start of infection and thus, failed to determine the stage of infection in patients.

During *B. microti* infection, different strains of mice display majority of symptoms noticed in human patients (Vannier et al., 2004; Coleman et al., 2005; Skariah et al., 2017). Therefore, the major objective of this study was to examine if *B. microti* infection cycle in TLR4 deficient C3H/HeJ mice follows the same pattern and assess the impact of parasitemia on the mammalian host pathology and immune response. Our qPCR result showed very high levels of parasitic DNA in blood of mice 21 days post infection while examination of blood smears by microscopy demonstrated barely detectable parasitemia. A follow-up careful examination of slides indicated that although parasitized erythrocytes were not detected readily, released parasite representing various life stages of *B. microti* could be observed, thus explaining our qPCR results. These findings are similar to culture of *B. divergens* after growth synchronization by Cursino-Santos et al. (2016). *In vitro* culture of *B. microti* and *B. duncani* is not established yet (Skariah et al., 2017). Although we succeeded in *B. microti in vitro* culture for some passages when active parasites were used for infection, parasites in the absence of ring-forms in RBCs, depicting active *B. microti*, failed to infect erythrocytes. However, by reviewing blood smears in available literature, different life stages of *Babesia* species could be observed outside the erythrocytes (Healy and Ruebush, 1980; Shih et al., 1997; Kjemtrup and Conrad, 2000; Saito-Ito et al., 2000; Vannier et al., 2008; Sun et al., 2013). These extra-erythrocytic parasites could be mere remnants of passing parasites or exhibit still active infection. Infectivity of these parasites was not determined.

Involvement of spleen in resolution of babesiosis in humans is well established (Vannier et al., 2004; Coleman et al., 2005; Genda et al., 2016). In our experiments, spleens of all infected mice were significantly enlarged, likely due to its hyperactivity during *B. microti* infection because of increased hematopoietic support and macrophages activity resulting in erythrophagocytosis (Homer et al., 2000). In *B. microti* infected mice, various parasitic forms were distinguishable even in spleen sections stained with Hematoxilin-Eosin. Although parasitemia was undetectable by microscopy at 21 days of infection, detection of *B. microti* DNA by our qPCR supports possibility of continuing low-level infection. Our analysis of white blood cells populations in spleen at this time point showed that both CD19+ B, and CD3+ T cell populations were depleted significantly while proportion of macrophages increased. Despite subversion of splenic adaptive immune response, mice were able to clear *B. microti* parasitemia in blood. Supporting our results, contribution of macrophage in clearance of *B. microti* in mice has been noted previously (Terkawi et al., 2015).

To determine the existence of potentially active infection by *B. microti* during apparent convalescent period when certain level of immune response is maintained, we analyzed concentrations of plasmatic cytokines. We found higher concentration of IL-6, IFN-γ, and TNF-α that have been shown to be the major pro-inflammatory cytokines produced in response to *B. microti* infection (Igarashi et al., 1999; Hemmer et al., 2000b). Increased cytokines production during babesiosis in patients could be responsible for febrile illness and even myalgia. High concentrations of IL-4 and IL-10, likely produced by regulatory B cells, play a major role in disease resolution but could also indicate still active *B. microti* infection (Hemmer et al., 2000a; Jeong et al., 2012). In fact, production of anti-inflammatory IL-10 in *B. microti* infected mice was suggested to be important for survival of the host (Hemmer et al., 2000a). Increase in cytokines levels agree with our qPCR results that detected parasites in blood. Thus, inability to detect parasitemia in patients based upon infected RBCs presence appears to erroneously leave out infectious but released and free *B. microti* parasites. Thus, our studies follow the infection cycle of *B. microti* and provide both direct (qPCR and microscopy) and indirect (increase in the specific plasmatic cytokines) evidence of active infection in host despite apparent resolution of babesiosis as detected by parasitemia measurement.

Involvement of CD4 T cells, and IFNγ cytokine has been shown to confer protective immunity against various protozoan pathogens during mammalian infection (Favre et al., 1997; Igarashi et al., 1999; Hemmer et al., 2000a; Hoft et al., 2000; Wang et al., 2004; McCall and Sauerwein, 2010). Previous studies showed that although not essential, CD4 T cells and IFNγ also play important roles in protective immunity against *B. microti* infection in mice (Igarashi et al., 1999; Hemmer et al., 2000a,b; Skariah et al., 2017). Significantly high levels of IL-2, TNFα, and IFNγ in infected mice could be produced by activated CD4 helper (Th), primarily Th1 cells potentially in response to antigens of *B. microti* they encounter, indicating that cell mediated immunity is very important for clearance of the parasites from blood. Previously reported persistence of *B. microti* parasitemia in CD4-/- mice supports this hypothesis (Skariah et al., 2017). Increase in the plasmatic levels of IL-4, IL-5, and IL-13 produced by Th2 cells was not as significant. These results suggest relatively less critical role of humoral immunity in resolution of babesiosis because Th2 cells support stimulation of antibody production. Interestingly, the levels of IL-6 and IL-10 cytokines, which are also produced by Th2 cells, were found to increase significantly and to a much higher levels in the infected mice. However, IL-6 and IL-10 are also produced by activated macrophage and B cells also produce IL-10 (Williams et al., 2000). High levels of IL-10 in serum of *B. microti*-infected mice have also been reported previously (Jeong et al., 2012). Increase of 5–10-fold levels in TNFα production in response to human *Babesia* WA1 strain later in infection, suggested to be produced by CD8+ T cells, and concomitant decrease in IL-10 levels was occurred together with fatal disease in mice (Hemmer et al., 2000a). We observed resolution of babesiosis caused by *B. microti* despite significant increase in plasmatic TNFα level but it was not as high as reported for WA1 strain, which could be attributed to simultaneous increased production of IL-10 and its anti-inflammatory activity. IL-10 may also suppresses overexpression of both IFNγ and TNFα while allowing enough immune response to build that is sufficient to eradicate *B. microti* infection (Trinchieri, 2007; Couper et al., 2008a,b; Redpath et al., 2014).

Our results further emphasize the importance of macrophage in immunity against *B. microti* infection. Significant increase in IL-6 that promotes differentiation of Th17 cells (McGeachy et al., 2007) was not found to be associated with downstream increase in cytokines production by Th17 cells, a unique CD4 cells. Th17 cells produce both IL-17 and IL-21, which are implicated in various inflammatory responses (Miossec et al., 2009). Unlike previous studies with *Helicobacter pylori* where infection causes adenocarcinoma and produces high levels of IL-17 and IL-21 (Amedei et al., 2014), only moderate increase in signature cytokines secreted by Th17 cells, such as IL-17, IL-17F, IL-21, and IL-22 (Volpe et al., 2008; Korn et al., 2009), was observed in our experiments. These results suggest that Th17 cells play only a minor role during *B. microti* infection and do not trigger a pronounced inflammatory response, as observed during *Babesia* WA1 infection. This is not surprising because Th17 cells usually are found to be more important players during diverse immune-mediated diseases. In summary, our studies confirm the roles of CD4 cells and macrophage in clearance of parasitized erythrocytes during *B. microti* infection and thus, contributing to resolution of babesiosis (Igarashi et al., 1999, #10007; Hemmer et al., 2000a,b; Aguilar-Delfin et al., 2003; Skariah et al., 2017).

## Conclusion

Our studies with patients and mice samples indicate that qPCR could be effective for detection of both parasitized erythrocytes as well as free parasites released after lysis of infected RBCs. We confirm that spleen is important for resolution of babesiosis in mammalian hosts. Furthermore, increase in pro-inflammatory plasmatic cytokines, IL-6, IFN-γ, and TNF-α together with the presence of activated macrophage in both blood and spleen could help in resolution of infection with *B. microti*.

## Author Contributions

All authors contributed significantly to this work. NP conceived the study and VD and LA designed and conducted all experiment. LA started this study and VD analyzed data and wrote first draft of the manuscript. All authors have read and approved the manuscript for submission.

## Conflict of Interest Statement

The authors declare that the research was conducted in the absence of any commercial or financial relationships that could be construed as a potential conflict of interest.

## Abbreviations

FISH: fluorescence *in situ* hybridization
IFA: indirect immunofluorescence assay
qPCR: real-time quantitative polymerase chain reaction
TTB: transfusion transmitted babesiosis
